# Association between physical exercise and stroke recurrence among first-ever ischemic stroke survivors

**DOI:** 10.1038/s41598-021-92736-5

**Published:** 2021-06-28

**Authors:** Lisha Hou, Mier Li, Ju Wang, Yawen Li, Qianwen Zheng, Lu Zhang, Qiang Yao, Jing Zhang, Shuju Dong, Muke Zhou, Cairong Zhu

**Affiliations:** 1 West China School of Public Health and West China Fourth Hospital, Sichuan University, No. 17 Section 3, Renmin South Road, Chengdu, 610041 Sichuan China; 2 National Clinical Research Center for Geriatrics, West China Hospital, Sichuan University, Chengdu, Sichuan Province China; 3 Department of Neurology, West China Hospital, Sichuan University, Chengdu, Sichuan China

**Keywords:** Diseases, Risk factors

## Abstract

The relationship between exercise and stroke recurrence is controversial. This study was designed to test whether an association exists between exercise and ischemic stroke recurrence in first-ever ischemic stroke survivors. Data were collected from January 2010 to June 2016. Baseline information was obtained during face-to-face interviews, and follow-up phone interviews were conducted every 3 months. Exercise type, frequency, intensity, and duration were recorded. Discrete-time survival analysis was used to determine the relationship between exercise and stroke recurrence. 760 first-ever ischemic stroke survivors who were able to exercise were enrolled. After adjusting for covariates, patients who exercised 3.5–7 h per week and more than 7 h per week had a lower relapse risk than patients who did not exercise (3.5–7: OR 0.415; > 7: OR 0.356). Moreover, if the fluctuation of exercise duration was over 4 h, the patients had a higher risk of stroke recurrence than those with variability of less than 2 h (OR 2.153, *P* = 0.013). Stroke survivors who engage in long-term regular mild exercise (more than 5 sessions per week and lasting on average 40 min per session) have a lower recurrence rate. Irregular exercise increases the risk of stroke recurrence.

## Introduction

Stroke is the second leading cause of death and the most frequent cause of disability in adults globally^1^. In 2010 alone, stroke resulted in 1.7 million deaths^2^ in China, indicating that more than 3 individuals die of stroke every minute. The rate of recurrence of ischemic stroke is high; it ranges from 16 to 29% in the USA^3^ and is calculated to be 29.43% in China^4^. The high incidence of recurrence increases the mortality^5,6^ and decreases the ability to compensate for the injury functionally^5^. It has been estimated that active prevention can reduce the rate of the recurrence of ischemic stroke by approximately 80%^7^.

High-quality studies have documented unequivocally that exercise reduces blood pressure^8,9^ and insulin resistance^10^, improves endothelial function^11^ and lipid metabolism^12–14^, and helps lose weight^15^. Additionally, physical exercise reduces inflammatory processes and expression of apoptotic markers, promotes angiogenesis in the brain, upregulated the expression of certain growth factors, and improves the activation of muscles involved in the exercise^16^.

Previous studies have suggested that physical activity is associated with a reduction of first stroke attack. A meta-analysis showed that active individuals had a 27% lower risk of stroke incidence or mortality (RR = 0.73) than low-active individuals^17^.A 10-year follow-up study of mortality revealed that high-physical-activity group had a lower risk of death from stroke than low-physical-activity group^18^. However, there is a lack of long-term follow-up investigations on the relationship between physical exercise and recurrent stroke^19–21^, and whether such the relationship exists remains uncertain. While some studies have concluded that physical exercise has no effect on recurrent stroke^22–25^, other^24,25^ have suggested that physical exercise protects against stroke recurrence. These studies^22–25^ ignored the long-term changes in physical exercise after stroke and their effects on relapse. Billinger and coworkers^19^ recommended that stroke survivors should engage in low- to moderate-intensity aerobic activity and muscle-strengthening exercises. However, the recommended intensity of physical activity in stroke survivors remains controversial. For example, the American Heart Association/American Stroke Association (AHA/ASA) advised at least 3 to 4 sessions per week of moderate- to vigorous-intensity aerobic exercise^26^. On the other hand, given that the stroke guidelines available in China do not address the behavioral lifestyle, Chinese stroke survivors are not informed about the necessity of proper exercise.

The present study is based on a cohort of prospective ischemic stroke survivors and provides a detailed analysis of long-term changes in physical exercise and the impact of post-stroke physical exercise on the recurrence of ischemic stroke.

## Materials and methods

### Study population

The study was approved by the Ethics Committee of the West China Hospital, Sichuan University, Chengdu, China. All patients had signed informed consent. We confirm that all methods were performed in accordance with relevant guidelines and regulations. A total of 764 patients with a primary and first diagnosis of ischemic stroke were recruited during the admission to the two medical groups in the Department of Neurology, West China Hospital, Sichuan University between January 2010 and June 2016. Ischemic stroke was diagnosed by a senior physician according to the World Health Organization definition^27^ and brain imaging results (CT scan and MRI). Considering medical disputes and compliance, the patients with iatrogenic stroke, such as carotid endarterectomy, cardiac surgery, or angioplasty, were excluded. Patients who were bedridden and unable to participate in physical exercise after discharge were also excluded.

### Study design

After obtaining written consent, trained interviewers used a semi-structured questionnaire in a face-to-face setting to obtain information on the basic characteristics and lifestyle of patients before the ischemic stroke. For patients who had cognitive or language impairment, proxies were interviewed. The questionnaire collected information on demographic characteristics, lifestyle, physiological and biochemical indexes, comorbidities, and medication history. Follow-up interviews were conducted by phone every 3 months after hospital discharge. The questionnaires collected information on rehabilitation, changes in lifestyle, ischemic stroke recurrence and treatment, comorbidities, medication history, and antithrombotic medication compliance. Information contained in patients’ medical records at the hospital was also retrieved.

### Physical exercise assessment

The widely used definition of physical activity and physical exercise was given by Caspersen and collaborators^28^: “Exercise is a subset of physical activity that is planned, structured, and repetitive and has as a final or an intermediate objective the improvement or maintenance of physical fitness.” In this study, we define exerciser as patients who exercise at least once a week. Patients who exercised less than once per week was classified as non-exerciser. The self-reported physical exercise questionnaire was used to collect data on the physical exercise of stroke survivors before and after the stroke. Four aspects of exercise were measured: type, frequency, intensity, and duration. The following questions were used in the questionnaire: “Have you exercised during the last 3 months?”, “What type of exercise did you do?”, “On average, how many times per week did you perform this activity?” and “On average, how many minutes each time?” The duration of exercise per week was calculated as the average during the entire follow-up period. Weekly exercise time fluctuations were defined as the standard deviation of physical exercise time per week.

### Recurrence measurement

The current study enrolled the survivors of the first-ever ischemic stroke. Terminal events included recurrent ischemic stroke, transient ischemic attack, and death resulting from recurrent ischemic stroke. Some patients have relapsed more than once (32/154), and the first relapse was chosen as the terminal event.

### Statistical analysis

The recurrence rate of stroke among the survivors and the relationship between physical exercise and recurrent stroke were assessed using discrete-time survival analysis^29–31^. This method was employed since the observation time of every three-month follow-up was discrete and the model can directly incorporate time-dependent variables. Exercise frequency and duration of exercise each week were entered into the model.

The possible confounding factors included age, gender, family history of stroke, marital status, education, occupation, stroke severity, total annual household income, lesion location, smoking, drinking, BMI, hormone medication history, antithrombotic medication compliance, and comorbidities. Age, gender, family history of stroke, marital status, educational attainment, smoking, drinking, and annual family income were self-reported. The patients’ age, gender, marital status, education, and occupation, as well as stroke severity and lesion location, were consistent with the data previously published by our group^32^. Stroke severity was assessed 3 months after hospital discharge using the modified Rankin Scale (mRS). Heart diseases included myocardial infarction, congestive heart failure, valvular heart disease, atrial fibrillation, and coronary heart disease. Hypertension, diabetes, heart disease, and peripheral vascular disease were assessed based on medical records. According to the Chinese “Healthy Adult Weight Determination” (WS/T 428-2013), BMI was categorized as underweight (< 18.5 kg/m^2^), normal weight (18.5–23.9 kg/m^2^), overweight (24–27.9 kg/m^2^) and obesity (≥ 28.00 kg/m^2^). Antithrombotic medications included aspirin, clopidogrel, and warfarin. Categorical variables were entered as dummy variables. The pre-treatment and analysis of the data were mostly conducted in SAS 9.3 software (SAS Institute Inc., Cary, NC, USA).

## Results

### Demographics

**Figure 1:**
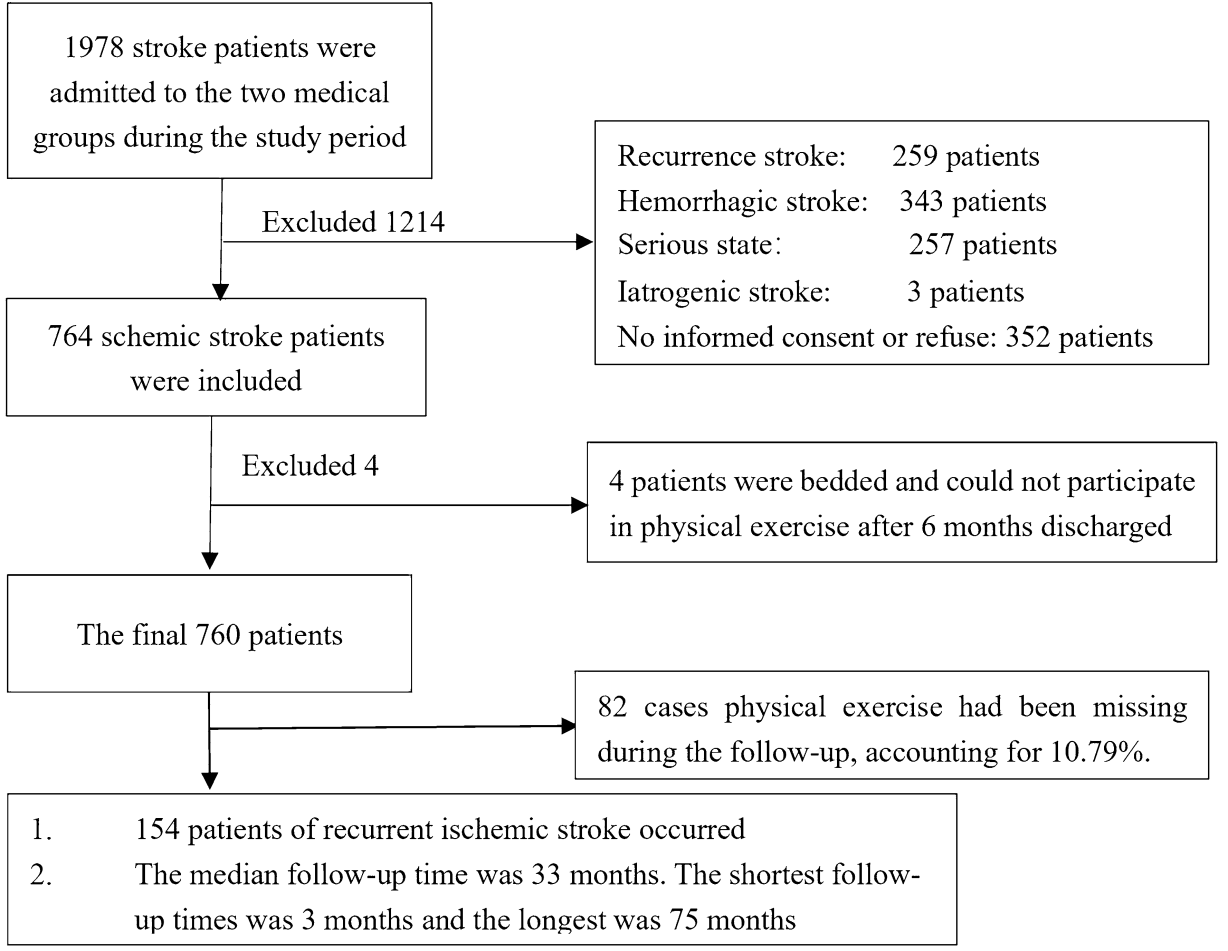
Flow of study participants.

A total of 764 first-ever ischemic stroke patients were recruited from January 2010 to June 2016. Of those, 760 patients were capable of participating in physical exercise and were eventually included in the study (Fig. 1). Among them, 462 were male (60.87%) and 298 were female (39.21%). At baseline, the subjects' age ranged from 17 to 90 years, with a mean age of 61.97 ± 12.69 years.

### Recurrence of ischemic stroke in survivors with different characteristics

Table 1 summarizes the recurrence of ischemic stroke in patients with different demographic and medical characteristics (censored at follow-up is not listed as recurrent). Patients of older age, lower income, other side of lesion location, history of heart disease, and non-compliant patients had a higher recurrence rate (*P* < 0.05).

**Table 1:** Recurrence of survivors with different characteristics.

Characteristics	Recurrence status N (%)		*χ*^*2*^	*P*
	No recurrence (N = 606)	Recurrence (N = 154)		
**Gender**			2.536	0.111
Male	377 (81.60)	85 (18.40)		
Female	229 (76.85)	69 (23.15)		
**Age**			16.390	< 0.001
< 45	87 (94.57)	5 (5.43)		
45 ~ 64	264 (80.00)	66 (20.00)		
≥ 65	255 (75.44)	83 (24.56)		
**Family history of stroke**			1.215	0.270
No	483 (78.92)	129 (21.08)		
Yes	122 (82.99)	25 (17.01)		
**Marital status**			2.248	0.134
No spouse	71 (73.96)	25 (26.04)		
With spouse	534 (80.54)	129 (19.46)		
**Education**			3.240	0.198
≤ 6 years	218 (76.22)	68 (23.78)		
7 to 9 years	157 (81.77)	35 (18.23)		
≥ 10 years	226 (81.59)	51 (18.41)		
**Occupation**			7.581	0.056
Farmer	150 (78.13)	42 (21.88)		
Not farmer	160 (86.49)	25 (13.51)		
Retired	241 (76.51)	74 (23.49)		
Unemployed	54 (72.92)	13 (27.08)		
**Total annual household income**			8.738	0.013
< $1500	190 (74.80)	64 (25.20)		
$1500 ~ $6000	215 (79.34)	56 (20.66)		
≥ $6000	201 (85.53)	34 (14.47)		
**Lesion location**			19.590	< 0.001
Left	191 (84.51)	35 (15.49)		
Right	160 (83.77)	31 (16.23)		
Bilateral	81 (84.38)	15 (15.63)		
Other	174 (70.45)	73 (29.55)		
**Hypertension**			0.489	0.485
No	231 (81.05)	54 (18.95)		
Yes	375 (78.95)	100 (21.05)		
**Diabetes**			0.115	0.735
No	445 (80.04)	111 (19.96)		
Yes	161 (78.92)	43 (21.08)		
**Hyperlipidemia**			0.003	0.953
No	393 (79.88)	99 (20.12)		
Yes	212 (79.70)	54 (20.30)		
**Heart disease**			5.679	0.017
No	483 (81.59)	109 (18.41)		
Yes	123 (73.21)	45 (26.79)		
**Peripheral vascular disease**			0.916	0.339
No	590 (79.95)	148 (20.05)		
Yes	15 (71.43)	6 (28.57)		
**Hormone medication history**			–	0.072
No	285 (81.20)	66 (18.80)		
Yes	12 (63.16)	7 (36.84)		
**Smoking**			3.210	0.360
No	258 (77.01)	77 (22.99)		
Passive smoking	93 (79.49)	24 (20.51)		
Quit smoking	204 (82.59)	43 (17.41)		
Yes	45 (83.33)	9 (16.67)		
**Drinking**			1.085	0.298
No	390 (78.63)	106 (21.37)		
Yes	216 (81.82)	48 (18.18)		
**BMI**			0.402	0.940
Underweight	39 (78.00)	11 (22.00)		
Normal	294 (79.25)	77 (20.75)		
Overweight	204 (80.95)	48 (19.05)		
Obesity	66 (80.49)	16 (19.51)		
**mRS**			2.325	0.127
< 3	216 (82.76)	45 (17.24)		
≥ 3	388 (78.07)	109 (21.93)		
**Antithrombotic medication compliance**			9.924	0.002
Compliance	257 (83.99)	49 (16.01)		
Non-compliance	273 (73.98)	96 (26.02)		

### Changes in physical exercise in ischemic stroke survivors

During the year preceding the ischemic stroke, only 42.37% of patients engaged in physical exercise. Patients who were male, younger, lower education, lower income, smoker, drinker and worse functional condition were less likely to exercise (*P* < 0.05). There were 15 types of physical exercise (such as Walking, Dancing Running, Gymnastics, Equipment training and Tai Chi etc.). Walking (78.57%) was the most type of exercise activity, followed by dancing (5.59%) and running (5.28%). Most patients (91.93%) only participate in one type of physical exercise, and 71.12% of patients only participate in walking. According to the metabolic equivalent (MET) values (low intensity: MET < 3; medium intensity:3 ≤ MET ≤ 6; high intensity: MET > 6), 91.93% participated in low and medium intensity physical exercise.

After stroke, the proportion of patients performing physical exercise was 60% or more. Approximately 90% of patients performed only one type of physical exercise, and only 5–8% of patients participated in two types of exercise. Only one patient participated in three distinct physical exercises during the entire follow-up period. More than 85% of patients participated in group walking as the form of physical exercise. The data indicate that walking was the primary way of exercising among ischemic stroke survivors. Considering that these patients practice a single form of low-medium-intensity physical exercises, the type and intensity of exercise were not included in further analysis, only the frequency duration of the exercise was considered.

### Changes in frequency and duration of physical exercise in ischemic stroke survivors

**Figure 2:**
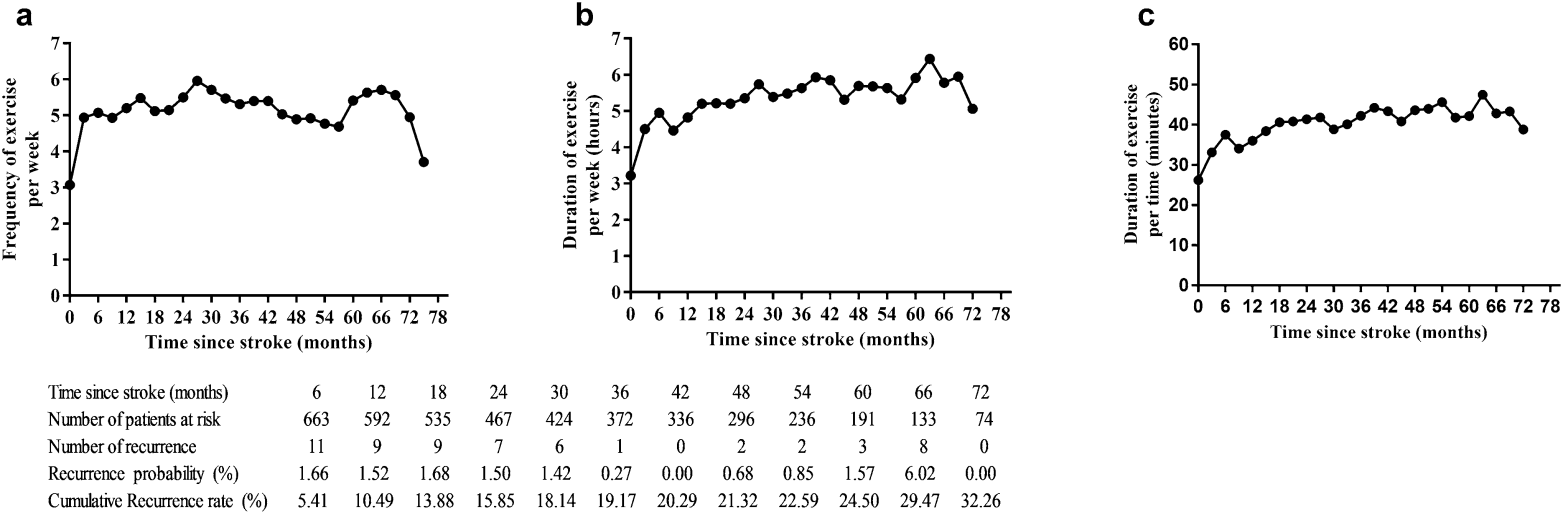
Changes in physical exercise before and after ischaemic stroke.

Figure 2 displays the long-term changes in exercise before and after ischaemic stroke. The general trend in average exercise time and exercise frequency showed increases, increasing from an average of 3.07 times to 5–6 times per week, from an average of 3.22 h to nearly 6 per week and from an average of 26.24 min to nearly 40 min per time, respectively.

### Effect of physical exercise on stroke recurrence

**Figure 3:**
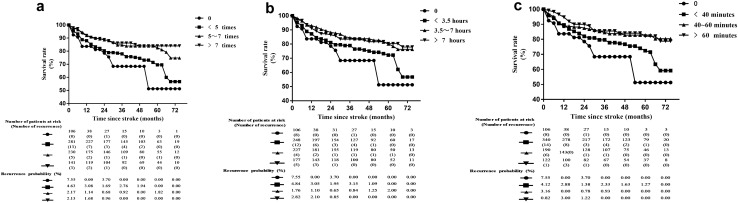
Survival curve of stroke survivor for different physical exercise characteristics.

During the long-term follow-up, 154 subjects (20.26%) relapsed. The cumulative recurrence rate at 3 and 6 months, and 1, 2, 3, 4, 5, and 6 years were, respectively, 3.82%, 5.41%, 10.49%, 15.85%, 19.17% 21.32%, 24.50%, and 32.26%. The survival curve in patients with different exercise characteristics is shown in Fig. 3. Patients who did not exercise had the highest rate of stroke recurrence.

### Association between stroke recurrence and physical exercise

**Figure 4:**
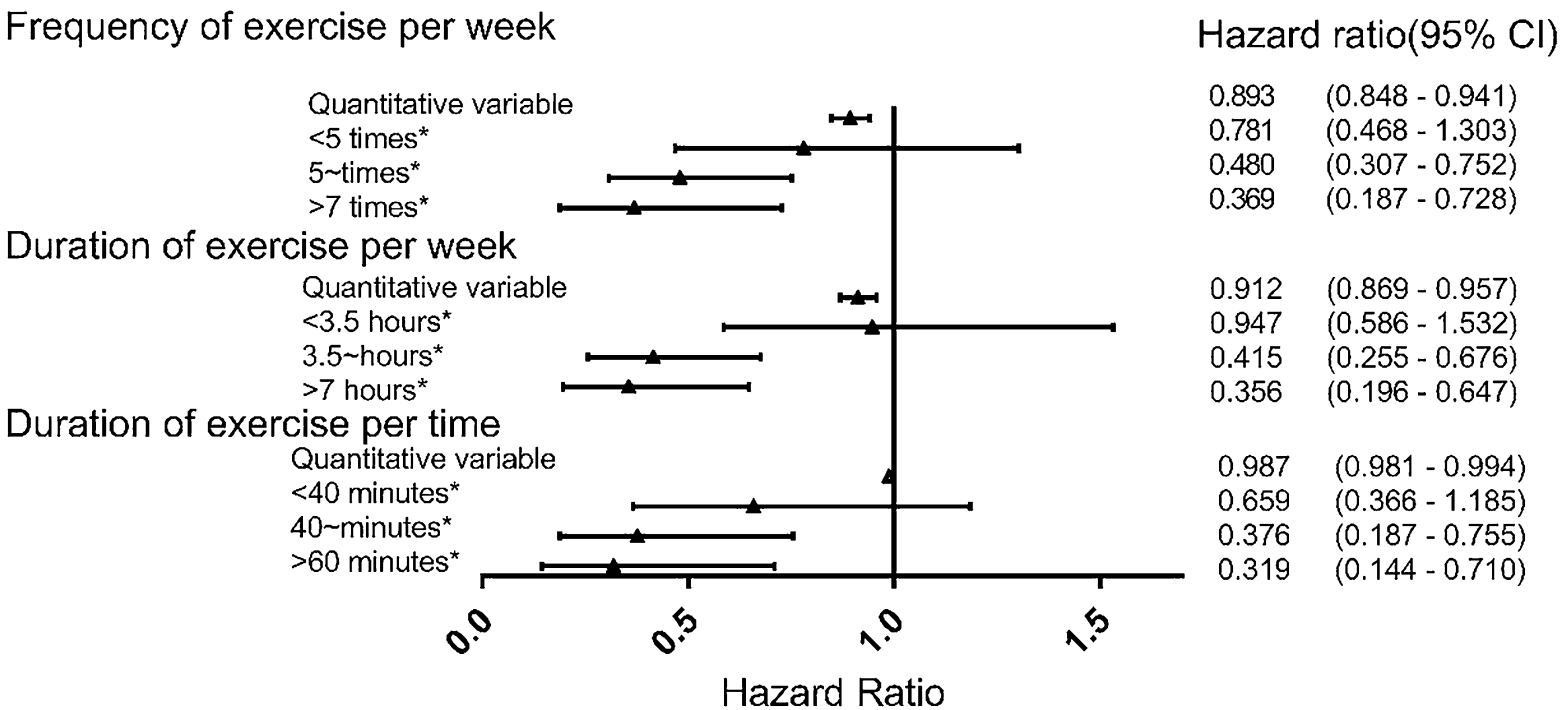
The effect of exercise on stroke recurrence.

First, the number of physical exercise per week was included in the model as a continuous variable. A statistically significant difference in the risk of recurrence of stroke was present between the patients engaging in different numbers of weekly physical exercises (*P* < 0.001) and each additional exercise per week reduced the risk of stroke recurrence by 10.7% (Fig. 4). Subsequently, the number of exercise activities per week was included in the model as a categorical variable. The risk of relapse in non-exercising patients was higher than in those who exercised 5 to 7 times per week (OR 0.480, *P* = 0.001) and those who exercised more than 7 times per week (OR 0.369, *P* = 0.004).

Adjusted hazard ratios (and 95% CI) associated with frequency of exercise per week, duration of exercise per week, duration of exercise per time, for the outcome recurrent ischaemic stroke and TIA.

The horizontal lines are 95% confidence limits.

Gender, age, marital status, education, occupation, income, lesion side, hypertension, diabetes, hyperlipidemia, heart disease, peripheral vascular disease, smoking, drinking, BMI, mRS, medication compliance and family history of stroke were adjusted.

Asterisk indicates that the reference group is that does not participate in physical exercise.

Figure 4 shows also the effects of the duration of physical exercise per week on the recurrence of ischemic stroke. A statistically significant correlation was present between the risk of stroke recurrence and duration of weekly exercises (*P* = 0.0002), and each additional 1 h of exercise per week reduced the risk of recurrent stroke by 8.8%. The risk of relapse in non-exercising patients was higher than those who exercised 3.5 to 7 h per week (OR 0.415,* P* = 0.0004) and those who exercised more than 7 h per week (OR 0.356, *P* = 0.001).

Finally, Fig. 4 illustrates the effects of the duration of physical exercise on the recurrence of ischemic stroke. The risk of stroke recurrence was significantly correlated with exercise time (*P* < 0.001) and each additional 1 min of exercise yielded a 1.3% reduction in the risk of recurrent stroke. The risk of relapse in non-exercising patients was higher than in those that exercised 40–60 min each time (OR 0.376,* P* = 0.006) and those that exercises more than 60 min (OR 0.319, *P* = 0.0005).

### Impact of regular physical activity on ischemic stroke recurrence

The current analysis used the standard deviation of the duration of physical exercise per week for each subject as a measure of exercise regularity. After controlling for other covariates, the regularity of each physical activity during the entire follow-up period for each patient was entered into the model. Data listed in Table 2 document that the average weekly exercise time was significantly different between groups and non-exercise groups (*P* < 0.05). When the fluctuation of exercise duration was over 4 hours, the patients had a higher risk of stroke recurrence than those with variability of less than 2 hours (OR 2.153, *P* = 0.013).

**Table 2:** The influence of regular exercise of stroke survivors on recurrence.

Variable	*β*	*P*	*OR*	95% *CI*
**Average duration of weekly exercise (No exercise)**				
< 3.5 h	− 0.750	0.028	0.472	(0.242, 0.922)
3.5–7 h	− 1.551	< 0.001	0.212	(0.095, 0.474)
> 7 h	− 1.803	< 0.0001	0.165	(0.067, 0.403)
**Weekly exercise time fluctuations (< 2 h)**				
2–4 h	0.093	0.729	1.098	(0.648, 1.860)
> 4 h	0.767	0.013	2.153	(1.174, 3.946)

After adjusted gender, age, marital status, education, occupation, income, lesion side, hypertension, diabetes, hyperlipidemia, heart disease, peripheral vascular disease, smoking, drinking, BMI, mRS, medication compliance and family history of stroke.

## Discussion

The results obtained in the present study demonstrate that the proportion of people participating in physical exercise has increased after ischemic stroke. However, the type of physical exercise appears monotonous, and its most common form is walking. Long-term, regular, mild, and low-intensity physical exercises were protective against stroke recurrence.

The awareness of the importance of physical exercise was increased in post-stroke patients, and 60% of them participated in these activities after the initial ischemic stroke. This proportion is still low when compared with the data presented by Astin and colleagues^33^ who have found that the fraction of patients participating in physical exercise increase from 52% before the stroke to 81% after the injury.

Among the patients enrolled in the current investigation, walking was the most common form of exercise, with more than half of the subjects engaging in this activity. This finding is consistent with a previous study^34^. It is recommended that stroke survivors should have an individualized activity plan that includes aerobic, strength, and flexibility exercises, developed in consultation with a health professional^35,36^. Most subjects in the present research chose walking as the form of exercise because they were not familiar with appropriate physical exercises after the stroke and might not have enough energy to participate in other types of physical effort. It was also reported that a third of stroke patients in the UK do not receive the necessary guidance on physical exercise^36^.

The results collected here demonstrate that low-intensity physical exercise protects against the recurrence of stroke and, in agreement with previous studies^34,37^ a dose–response relationship is present between these two variables. Sacco and coworkers^34^ identified a dose–response relationship between the duration of physical activity and the recurrence of stroke. Wannamethee and collaborators^37^ documented that low-intensity and moderate exercise reduce the risk of recurrent ischemic stroke and suggested that moderate activity, such as walking, should be encouraged. Conversely, the study by Kono and colleagues^23^ of 102 ischemic stroke patients in Japan concluded that low-intensity physical activity tended to be associated with higher recurrence. This notion is inconsistent with the result of the present work, and the discrepancy may be related to the difference in research design and the accepted definition of physical exercise.

The current investigation demonstrated that irregular exercise increases the risk of recurrent stroke. Although there is no direct evidence of the mechanism linking regularity of physical exercise and stroke recurrence, some insights can be gained from related studies. Regular exercise induces genome-wide epigenetic modifications in human skeletal muscle and adipose tissue. These modifications are reflected in altered mRNA expression, which can affect metabolic phenotypes and the risk of disease^38^. Radak and coworkers^39^ documented that regular exercise decreases the incidence of diseases related to the high generation of reactive oxygen species. Regular exercise by stroke survivors reduces the risk of recurrence by lowering the indices of obesity^40^. Therefore, it is essential to emphasize to the patients the importance of regular exercise after they are discharged from the hospital.

Generally, diabetes is a strong predictor for stroke recurrence. However, our results indicated that diabetes was not a predictive factor of recurrence (*P* > 0.05). Of the 760 patients, 204 (26.84%) patients had diabetes mellitus, of which 43 patients had recurrence (21.08%), 556 patients had no diabetes mellitus, and 11 patients had recurrence (19.96%), but the difference was not statistically significant. There may be two reasons: one reason was that the proportion of drug withdrawal and dressing change in diabetic patients was very small (drug withdrawal: 0.98–10.29%; dressing change: 1.96–12.25%) during each follow-up. It can be seen that the patients with diabetes had good compliance with diabetes drugs in this study, which was good for the prognosis of diabetes. Another reason was that the compliance with stroke among diabetic patients (52.49%) was better than non-diabetic patients (42.71%). The results of this study showed that drug compliance had an impact on recurrence (*P* < 0.05). The patients with poor drug compliance with stroke had a greater risk of recurrence.

The follow-up period in the present study was relatively long with a median time of 33 months (range, 3 to 75 months). Thus, the secular trend of physical exercise of the subjects could be analyzed, which enables us to gain a more comprehensive understanding of the characteristics of exercise in stroke survivors and to utilize fully the information on the changes in post-stroke physical activities to determine its impact on stroke relapse. Additionally, a time-dependent variable can be directly incorporated into the discrete-time risk model.

Some limitations of the present study should be acknowledged. The information on physical exercise was self-reported, which might negatively affect its accuracy. The habits of physical exercise were assessed every 3 months based on retrospective questionnaires, making the data prone to memory bias. The rate of stroke recurrence might have been underestimated since some patients might have experienced a transient ischemic attack without recognizing it. Furthermore, 32 of the 760 enrolled patients had stroke recurrence more than once, but considered only the first relapse. Finally, the study was lack of the information on ischemic stroke etiology and clinical features for classification of the subtypes of stroke were insufficiently collected at baseline. Neither the TOAST nor Oxfordshire Community Stroke Project classification of the subtypes of stroke was obtained, stroke lesion was included into the model as confounding factor.

To address these limitations in future research, an effort will be made to find more accurate, cost-efficient, and convenient methods to conduct the survey and to including a larger population of patients from different hospitals.

## Conclusion

Walking was the most common form of exercise among the survivors of ischemic stroke, with more than half of the patients engaging in this type of activity. Low-intensity physical exercise had a protective effect on stroke recurrence, and there was a dose–response relationship between the two variables. Irregular exercise increased the risk of recurrence of the stroke.

## Data Availability

The analysed data in the manuscript is available upon request from the corresponding author.
